# Effect of STAT3 inhibitor in chronic myeloid leukemia associated signaling pathway: a mathematical modeling, simulation and systems biology study

**DOI:** 10.1007/s13205-015-0357-7

**Published:** 2016-01-27

**Authors:** Himansu Kumar, Swapnil Tichkule, Utkarsh Raj, Saurabh Gupta, Swati Srivastava, Pritish Kumar Varadwaj

**Affiliations:** Indian Institute of Information Technology, Allahabad, 211012 India

**Keywords:** HGF, IL6, ODE’s, JAK/STAT and MAPK pathways

## Abstract

Chronic myeloid leukemia (CML) is a hematopoietic stem-cell disorder which proliferates due to abnormal growth of basophil cells. Several proangiogenic molecules have been reported to be associated in CML progression, including the hepatocyte growth factor (HGF). However, detail mechanism about the cellular distribution and function of HGF in CML is yet to be revealed. The proliferation of hematopoietic cells are regulated by some of the growth factors like interleukin 3 (IL-3), IL-6, erythropoietin, thrombopoietin, etc. In this study IL-6 pathways have been taken into consideration which induces JAK/STAT and MAPK pathways to decipher the CML progression stages. An attempt has been made to model these pathways with the help of ordinary differential equations (ODEs) and estimating unknown parameters through *fminsearch* optimization algorithm. Some of the specific component like STAT3, of the pathway has been analyzed in detail and their role in CML progression has been elucidated. The roles of STAT3 inhibitors into the treatment of CML have been thoroughly studied and optimum concentration of the inhibitors have been predicted.

**Electronic supplementary material:**

The online version of this article (doi:10.1007/s13205-015-0357-7) contains supplementary material, which is available to authorized users.

## Introduction

**Fig. 1:**
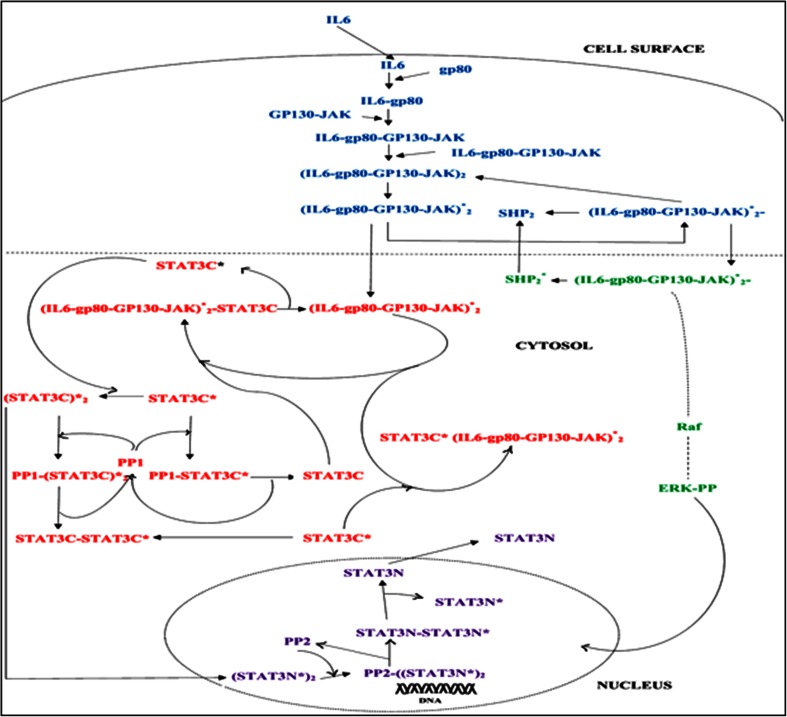
Schematic representation of JAK/STAT and MAPK Pathway, *blue color* indicates the common IL-6 pathway, *red* indicates the JAK/STAT, *Green* indicates the MAPK pathway, and *Magenta* indicates the nucleolar components

Chronic Myeloid Leukemia (CML) is a stem cell disorder resulted by abnormal growth of granulocytes (Strife and Clarkson 1988). The CML progression can be further differentiated into three phases namely Chronic, Accelerated and Blast depending on its severity, respectively (Kantarjian et al. 2006). The occurrence of CML has been reported to increase in two folds during last decade (An et al. 2010). It has been further reported that, translocation of the ABL gene on chromosome 9th to BCR gene on chromosome 22nd results into a hybrid Philadelphia chromosome with chimeric BCR-ABL genes (Daley et al. 1990; Rowley 1973; Ye et al. 2014). These chimeric genes encode BCR-ABL oncoproteins which contains the activated tyrosine kinase region of ABL, causing aberrant tyrosine kinase activity and hence promotes the proliferation of CML cells (National Comprehensive Cancer Network Clinical Practice Guidelines in Oncology, Chronic Myelogenous Leukemia, v2, 2009). The constitutive proliferations of BCR-ABL genes and their aberrant expressions lead to several abnormalities in the peripheral blood and bone marrow which consequently alters the number of granular leukocytes (Daley et al. 1990; Baccarani et al. 2006; Cerny-Reiterer et al. 2012). It is being reported that the earlier treatments processes involving chemotherapy, bone marrow and stem cell transplantation are often associated with serious side effects (Baccarani et al. 2006). Nowadays, CML is being treated by the BCR-ABL tyrosine kinase inhibitors, which leads to the increased rate of patient survivability. Most of inhibitors target the inhibition of ATP binding process with tyrosine kinase domain of ABL protein (Cerny-Reiterer et al. 2012; Zhang et al. 2010). The breakthrough treatment for CML was reported with the discovery of Imatinib (Savage and Antman 2002; Pray 2008). However, the reoccurrence of the CML cases in Imatinib treated patients has been reported due to the point mutation of amino acid residues in BCR-ABL receptors active site. Although few other receptors like STAT3, STAT5, and SRC have also been investigated as alternative to overcome the Imatinib resistance. In this study STAT3 receptor protein has been taken into consideration to explore the CML associated biochemical pathways like IL-6, which induces the MAPK and JAK-STAT (Reynaud et al. 2011; Bhalla and Iyengar 1999). IL-6 is an interleukin which is encoded by the IL-6 gene and responsible for pro-inflammatory cytokine and anti-inflammatory myokine activity. In overall IL-6, JAK/STAT and MAPK pathways, common gp130 (glycoprotein 130) binds to the plasma membrane receptor complex. Signal transduction activates the JAK tyrosine kinase family members, which activates the transcription factors of the STAT (Peifer et al. 2014). The interlinked pathway for IL-6-type cytokines is the MAPK pathway. Schematic representation of whole IL-6 associated signaling pathway is shown in Fig. 1. It has been reported that activation of STAT3 by bone marrow cells protects the CML from tyrosine kinase mediated inhibition of BCR-ABL protein. So, targeting the STAT3 in addition to BCR-ABL would enhance CML cells with kinase-independent resistance to TKI therapy. Recent study has reported BP-5-087 molecule as a multi-target inhibitor for BCR-ABL and STAT3 receptor. Further it was found that BP-5-087 molecule act on the Imatinib resistant cells and reduced the CML progression significantly (Eiring et al. 2015).

## Methods

The entire experiments were performed on Centos 6.5 of Linux operating systems with 12 GB RAM, NVIDIA graphics and 1 TB Hard disk computer system. Hardware of the system was as follows: Intel(R) Core (TM) i7-3770 CPU @ 3.40 GHz processor.

### Model description

CML associated signaling pathway in which IL-6 induces the two pathways, i.e. JAK/STAT and MAPK have been taken as model pathways. IL-6 is the first component of the said pathway, which enters into the cell by cell membrane and activates the entire pathway. In this process, IL-6 binds to the protein gp80, which is not involved in signaling process of the protein. The receptor gp130 which is a part of the signaling process attaches to the tyrosine kinases of JAK. Then IL-6-gp80 complex reacts to the compound gp130-JAK to form the complex IL6- gp80- gp130- JAK. Formation of this complex results in the dime formation of the complex IL6- gp80- gp130- JAK to form (IL6- gp80- gp130- JAK)2 (Heinrich et al. 2003). SHP-2 binds to the phosphorylated dimer complex (IL6-gp80-gp130-JAK)2 to form (IL6-gp80-gp130-JAK)*2-SHP-2 complex which act as the point of initiation for both the MAPK and JAK-STAT pathway (Yamada et al. 2003). In JAK-STAT signaling process, phosphorylated dimer (IL6-gp80- gp130-JAK)*2 recruits the transcription factor STAT3. The phosphorylation of the component STAT3 leads to its dissociation from the (IL6-gp80-gp130-JAK)2 complex and further undergo the process of dimerization. The dimerized complex of the STAT3 enters into the nucleus to undergo dephosphorylation and transferred back again into the cytosol for the another cycle of (Heinrich et al. 2003).

It has been reported that apart from BCR-ABL, STAT3 receptor also plays major role in CML progression which is an important component in JAK/STAT pathway. We assumed the initial concentration of STAT3 in the pathway to be 1 nM as earlier reported (Charusanti et al. 2004). From the literature, it is been reported that BP-5-087 can inhibit the activity of STAT3 by 70 %, therefore we took the inhibited (treated) concentration of STAT3 as 0.3 nM (Eiring et al. 2015). JAK/STAT signal transduction pathway is regulated by the two phosphatases of STAT3 namely PP1 and PP2. Theses phosphatases enhance the activation of STAT3 component in nucleolus and cytosol both. The phosphatase PP2 present in the nucleus is a very important part of JAK-STAT since it is responsible in deactivating the phosphorylated dimer complex of STAT3 in the nucleus. This process of deactivation, play a crucial role for coming back of STAT3 to the cytosol so that it can undergo another cycle of phosphorylation–dephosphorylation. Binding of SHP-2 to the complex (IL6- gp80-gp130-JAK)*2 plays a major role in linking JAK-STAT to the MAPK pathway. This process of complex formation is an important process for the MAPK signaling pathway.

### Kinetics of biochemical reactions

Schematic representation of chemical process of the pathway is shown in Fig. 1. The whole chemical processes in the pathway are decomposed into elementary reactions. This reaction model contains 67 state variables and 111 unknown rate constants. It has been assumed that initial concentration of each component is 1 nM and experimental concentrations are taken from reported literature (Charusanti et al. 2004). The kinetic reactions of the pathway are provided in Appendix I (Online Supplementary Material).

### Mathematical modeling of JAK/STAT and MAPK pathway

**Fig. 2:**
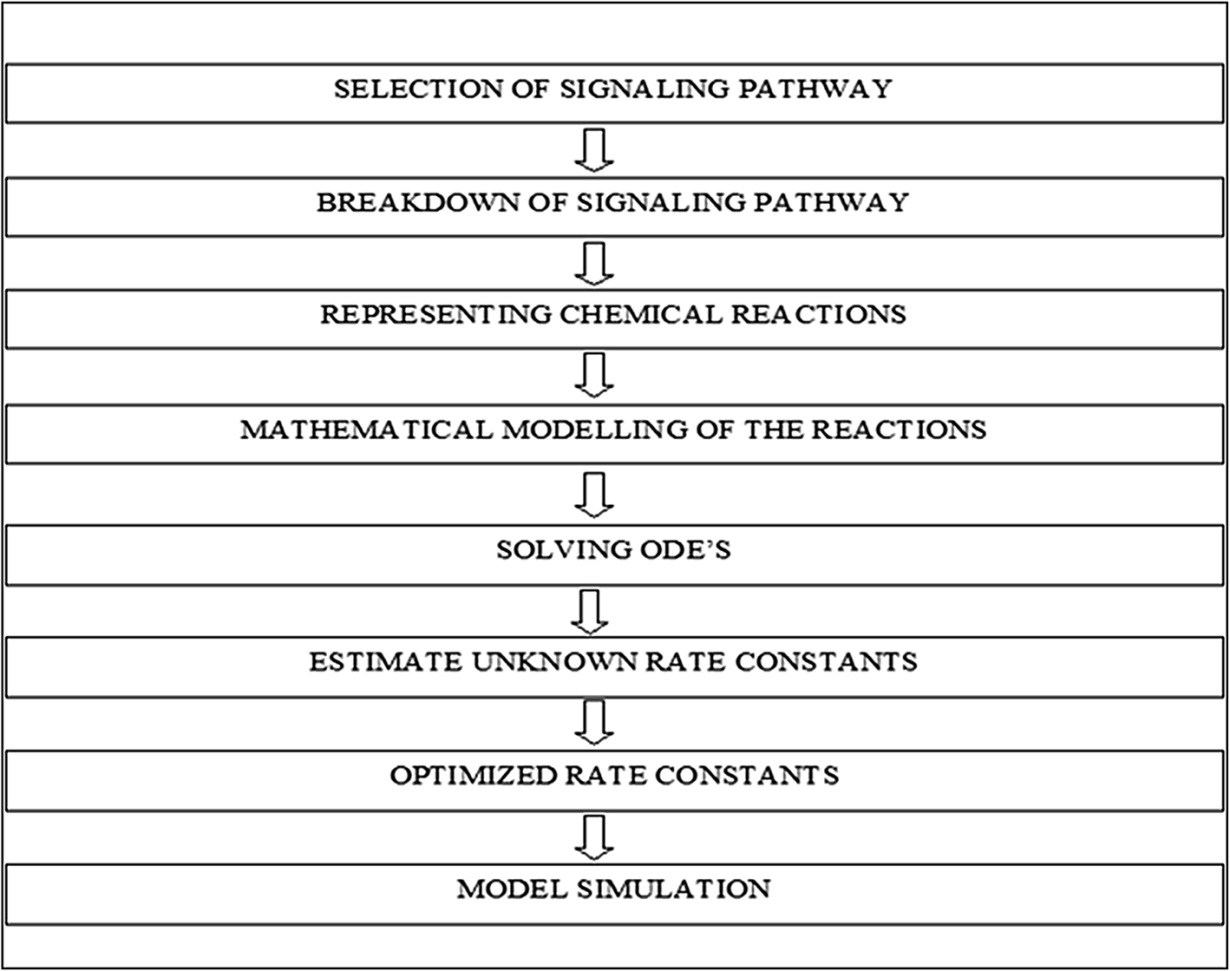
Flow chart of the adopted methodology

In this work, model parameters were calculated from the experimental data obtained from IL-6 induced signaling pathways. Systems biology techniques were used to understand various components involved in reaction mechanism of this pathway and to predict the behavior of individual reaction components (Heinrich et al. 2002; Papin et al. 2005; Tyson et al. 2003; Ge et al. 2013). Deterministic rate laws have been used to simulate various chemical and biochemical reactions (Edda et al. 2006; Tian and Song 2012; Klipp and Liebermeister 2006). The deterministic mathematical modeling of chemical network works on the basis of law of mass action and Michaelis–Menten reaction kinetics (Kanodia and Finn 2014). It has been further reported that the prior information of initial concentration, law of mass action etc., can enable the simulation of time series data to study substrate concentration and its temporal behavior (Edda et al. 2006; Tian and Song 2012). However, simulation process provides in depth understanding of each components of the biological systems and its activities in different environmental conditions (Gilbert et al. 2006; Guerriero et al. 2009). The rate of change in substrate concentration over a given period of time can be utilized to find the crucial parameters involved in the reaction mechanism (Salis and Kaznessis 2005). IL-6 induced signaling pathway has been simulated through deterministic mathematical modeling (Kwiatkowska et al. 2006). The candidate reaction pathways were simulated through ordinary differentiation equations to get the individual rate constant parameters (Deng and Tian 2014; Liepe et al. 2014). The parametric optimization was performed by “*fminsearch*” algorithm on MATLAB R2012a. Complete methodology has been shown in Fig. 2

In this process IL-6 induces two pathways like JAK/STAT and MAPK pathway which are responsible for various cellular processes.

### Parameter estimation

The parameters associated with above stated JAK/STAT and MAPK pathway’s reactions are estimated through ODE solver. This numerical simulation of model was performed by using MATLAB ODE solver toolbox (Bogacki and Shampine 1989). The standard ODE solver ODE45 was used to construct and solve the differential equations based on Runge–Kutta approximation method. Runge–Kutta methods involves implicit and explicit iterative methods for temporal discretization and approximation of ODE solutions. The proposed models with continuous states were being iterated and further optimized by *fminsearch.*

1 $$[t,x] = {\text{ode}}45( @ {\text{function, tspan}}, x0)$$

where ‘@function’ is the vector of function handles, ‘tspan’ a vector specifying the time span of simulation and ‘*x*0’ is the state variables of differential equations. The ‘tspan’ and ‘*x*0’ represents the specific time interval and initial starting conditions, respectively.

### Parameter optimization

*fminsearch* is a MATLAB function which minimize the error function with multiple variables, with the assigned estimated values of state variables. It utilizes nonlinear unconstrained multivariable optimization problems based on Nelder–Mead Sequential Simplex (NMSS) algorithm (Andrews and Larry 1992; Lagarias et al. 1998).

With the multiple functions are *f*(*x*1), *f*(*x*2)… *f*(*xn* + 1) the Nelder Mead Algorithm. NMSS is a nonlinear optimization technique, which is used to solve numerical problems with unknown derivatives. However, NMSS is a heuristic search method that can converge to non-stationary points (Powell 1973).

2 $$x = {\text{fminsearch}}( @ {\text{function}},x0)$$

where function is an M-file function such as

3 $${\text{function}}f = {\text{function}}(x)$$

4 $$f = {\text{fminsearch(@minimize}}, x0)$$

whereas, @minimize is a function containing objective function.

Error function used for minimizing scalar function is:

5 $${\text{Error}} = \mathop \sum \nolimits ({\text{Simulated\,result}}{-}{\text{Experimental\,data}})^{2}$$

### Experimental data

Experimental concentrations of the each substrate have been taken from (Singh et al. 2006) study. All the experimental values are shown in Appendix 1 (Online Supplementary Material) which is required for the modeling and simulation.

## Results and discussion

It has been reported that CML exhibit an aberrant expression of beta lymphoid cells which are directly influenced with cytokine IL-6 pathway, which involves two significant pathways like JAK/STAT and MAPK pathway. To get insight of the molecular mechanism of the CML, extensive in silico study of both pathways are needed. Aberrant expression of STAT3 in the IL-6 signaling pathway had been proven as prime cause of Chronic Myeloid Leukemia (CML). It has been established that STAT3 is a potential therapeutic target for the treatment of CML because it is an important component of the JAK/STAT pathway. Alteration or inhibition of STAT3 concentration indirectly influence the another associated MAPK pathway.

### Mathematical model of the JAK/STAT and MAPK pathway

These two interlinked pathways consist of 67 biochemical reactions and their 111 unknown rate constants. These biochemical reactions involve mass-action kinetic and Michaelis–menten kinetic reactions. All these reactions of the MAPK and JAK/STAT pathways were mathematically modeled in the form of ordinary differential equations. In continuation, all differential equations were solved by ode45 solver in MATLAB.

ODE models of the said chemical reactions are given in Supplementary material 3.

In this work role of STAT3 has been analyzed, as STAT3 is the most significant component of JAK/STAT pathway. STAT3 combines with (IL6- gp80- gp130-JAK) complex and form another complex, i.e. (IL6-gp80- gp130-JAK)-STAT3. STAT3 activates the (IL6- gp80- gp130-JAK) complex to enter into the MAPK pathway by dissociating themselves before the entry. By analyzing complete pathway of JAK/STAT and MAPK one can infer that STAT3 plays a decisive role into CML associated pathway.

### Parameter estimation

Experimental concentrations of the substrates involved in IL-6 induced signaling pathway are shown in Appendix I (Online Supplementary Material), Whereas ode45 solves the differential equation, *fminsearch* minimizes the error function and finds the optimized values of rate constants *kfi*, *kri, kmi* and, *kvi*. The simulation was initiated by considering unit value, i.e. 1 nM of each component of the reaction for a period of 32 min. After selection of random time intervals, 0–32 min gives the most closure value to the reported one. The results of simulation and values of unknown optimized rate constants are shown in Table 1.

**Table 1:** Estimated parameter values of the JAK/STAT and MAPK pathways

Parameter	Values	Parameter	Values	Parameter	Values
*kf1*	1.103424	*kr23*	1.106123	*kf45*	1.062902
*kr1*	0.947931	*k24*	1.012899	*kr45*	0.966483
*kf2*	1.035664	*k25a*	0.925016	*kf46*	1.021281
*kr2*	1.088271	*k25b*	0.964434	*kr46*	1.136333
*kf3*	0.967368	*k26*	1.011042	*kf47*	0.984223
*kr3*	0.861028	*k27*	0.962845	*kr47*	1.014038
*kf4*	1.035329	*kf28*	0.91007	*kf48*	1.009415
*kr4*	0.820722	*kr28*	0.959089	*kr48*	1.115897
*k5*	1.028834	*k29*	1.027849	*kf49*	1.075979
*kf6*	0.976114	*k30*	1.027844	*kr49*	0.945278
*kr6*	0.958236	*k31*	1.210422	*kf50*	1.096496
*k7*	1.022409	*kf32*	1.045764	*kr50*	0.981725
*kf8*	0.977993	*kr32*	0.93087	*k51*	1.043507
*kr8*	1.069307	*kf33*	1.04418	*kf52*	−0.30235
*kf9*	0.984565	*kr33*	0.901555	*kr52*	1.017269
*kr9*	1.056293	*kf34*	1.049215	*k53*	0.983402
*kf10*	1.009209	*kr34*	0.959882	*kf54*	1.000033
*kr10*	1.0096	*kf35*	0.99873	*kr54*	0.944497
*k11*	1.011066	*kr35*	0.959118	*k55*	1.216468
*kf12*	1.095124	*kf36*	0.976274	*kf56*	0.993236
*kr12*	0.975454	*kr36*	0.948192	*kr56*	1.059524
*k13*	0.96618	*kf37*	1.049747	*k57*	1.034902
*kf14*	1.008209	*kr37*	0.870539	*kf58*	1.056031
*kr14*	1.070989	*kf38*	0.978582	*kr58*	1.026759
*k15*	1.123849	*kr38*	1.011961	*k59*	1.062045
*kf16*	1.020288	*kf39*	1.028694	*kf60*	0.993215
*kr16*	0.959555	*kr39*	1.021497	*kr60*	0.953189
*k17*	1.010703	*kf40*	0.932659	*k61*	0.951944
*kf18*	1.001588	*kr40*	1.023355	*kf62*	1.012305
*kr18*	1.006706	*kf41*	0.96972	*kr62*	0.92763
*kf19*	1.217099	*kr41*	1.06176	*k63*	0.966868
*kr19*	1.002411	*kf42*	0.993106	*kf64*	1.024082
*k20*	1.168012	*kr42*	1.000562	*kr64*	1.002367
*kf21*	0.986824	*kf43*	1.010405	*k65*	0.965469
*kr21*	1.034464	*kr43*	1.083823	*kf66*	1.002329
*k22*	1.040881	*Vm*	0.991752	*kr66*	1.061648
*kf23*	1.0146	*Km*	1.045753	*k67*	0.972167

### Model simulation

The mathematical model of 67 state variables has been simulated with respect to time for a period of 32 min to understand the behavioral changes of the various components involved in the signaling pathway. The simulation results consists of both, the normal condition and the drug treated condition for all the components. The significant changes in the components have been reported in the following simulated results.

### STAT3 complexes

**Fig. 3:**
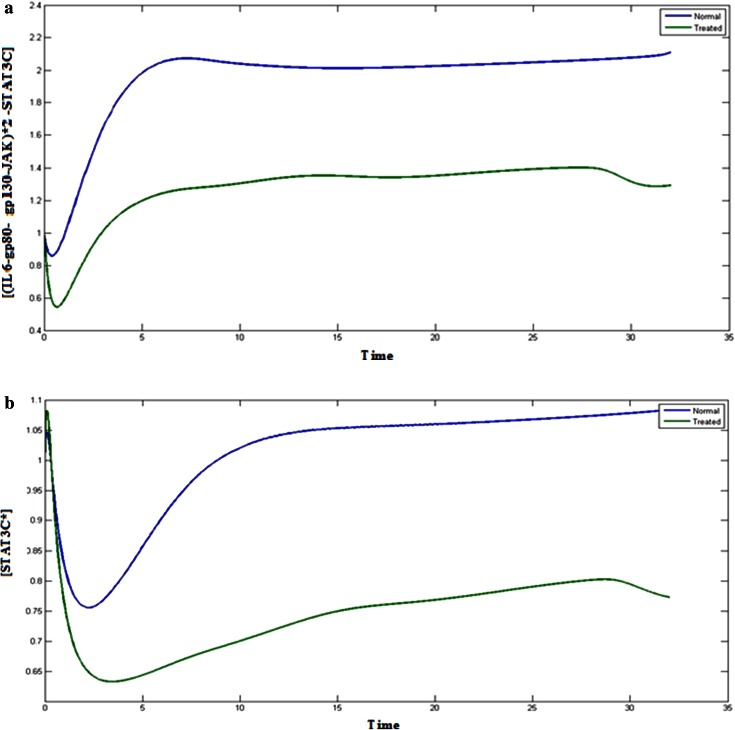
**a** *Blue color* (*upper*) indicates the normal cancerous cells and *green* (*lower*) treated cells, Plot of [(IL6-gp80- gp130-JAK)*2 -STAT3C] vs time. **b** *Blue color* (*upper*) indicates the normal cancerous cells and *green* (*lower*) treated cells, Plot of [STAT3C*] vs time

**Fig. 4:**
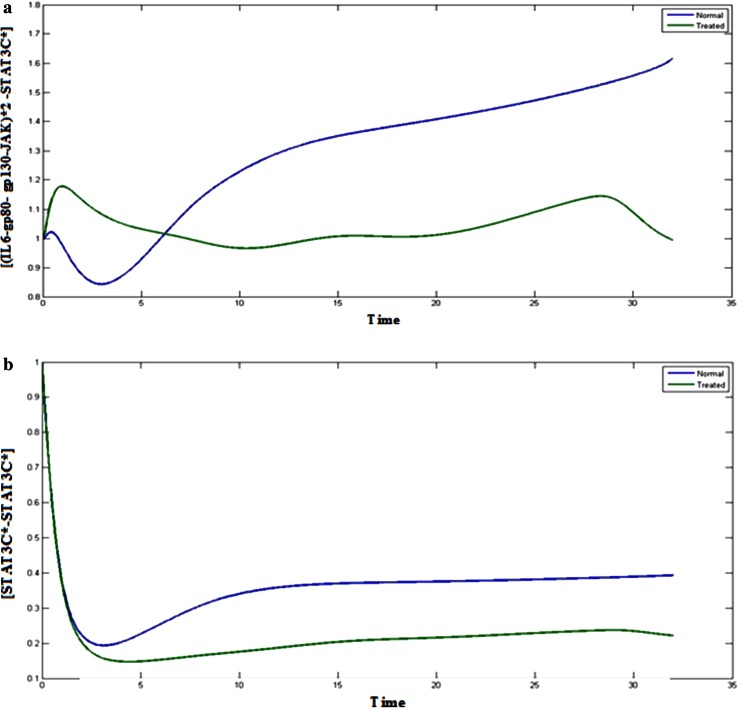
**a** *Blue color* (*upper*) indicates the normal cancerous cells and *green* (*lower*) treated cells, Plot of [(IL6-gp80- gp130-JAK)*2 -STAT3C*] vs time. **b** *Blue color* (*upper*) indicates the normal cancerous cells and *green* (*lower*) treated cells, Plot of [STAT3C*-STAT3C*] vs time

Existence of Cytosolic STAT3 is important for JAK/STAT signaling pathway which mainly involves the transcription factor, STAT3 for binding with various complexes in the pathway. If STAT3 is inhibited, it can hamper the signaling pathway through JAK/STAT and MAPK pathway. The Figs. 3a, b, and  4a, b represents the behavior of STAT3 complexes in normal condition and treated condition. Inhibition of STAT3 leads to decrease in the concentration of STAT3 complexes, such as (IL6-gp80- gp130-JAK)*2 -STAT3C, STAT3C*, (IL6- gp80- gp130- JAK)*2- STAT3C* and STAT3C*-STAT3C* as compared to the normal condition. Phosphorylation of STAT3 is essential for the signaling through JAK-STAT. These complexes help in the phosphorylation and transportation to the nucleus. If the STAT3 is inhibited, then it cannot be phosphorylated and consequently the JAK/STAT pathway will be inhibited.

(IL6-gp80- gp130-JAK)*2 –SHP2 acts as an interlinker between MAPK and JAK/STAT pathway. SHP-2 plays a crucial role in interlinking the JAK/STAT and MAPK pathways. Inhibition of STAT3 also affects the SHP-2 component. After binding of SHP-2 to (IL6-gp80- gp130-JAK)*2 complex, the level of (IL6-gp80- gp130-JAK)*2 –SHP2 got reduced.

### SHP-2 and its complexes

**Fig. 5:**
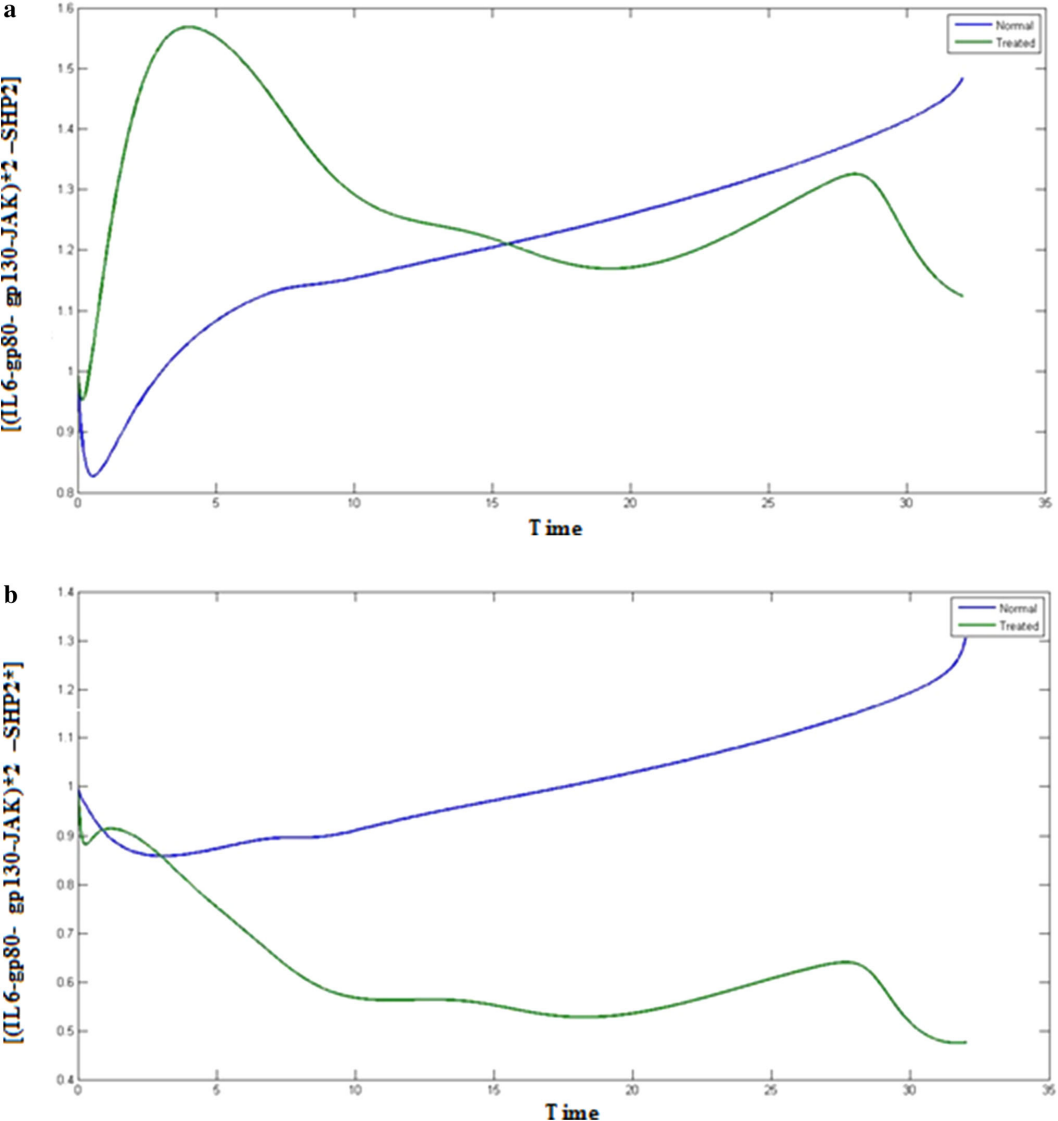
**a** *Blue color* (*upper*) indicates the normal cancerous cells and *green* (*lower*) treated cells, Plot of [(IL6-gp80- gp130-JAK)*2 –SHP2] vs time. **b** *Blue color* (*upper*) indicates the normal cancerous cells and *green* (*lower*) treated cells, Plot of [(IL6-gp80- gp130-JAK)*2 –SHP2*] vs time

**Fig. 6:**
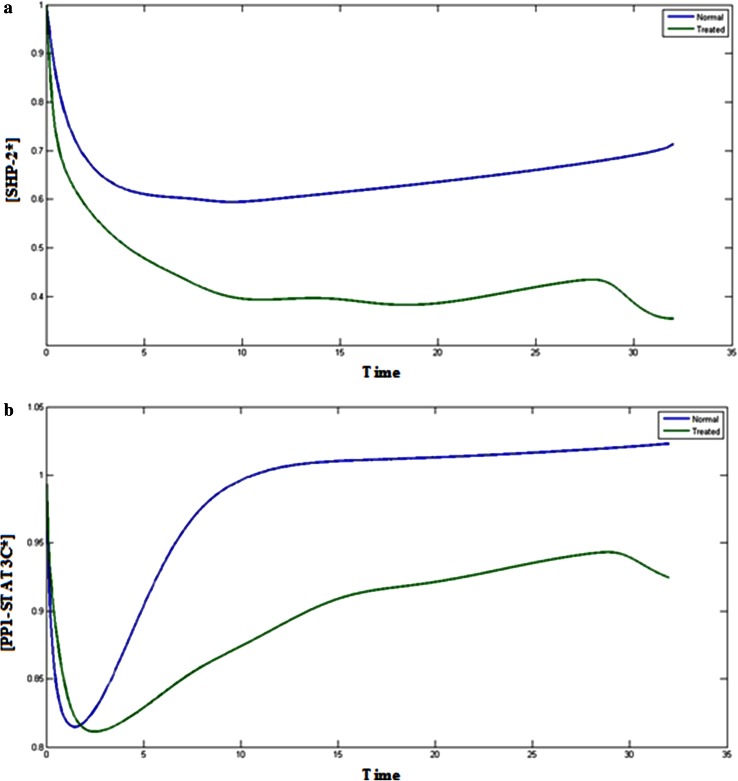
**a** *Blue color* (*upper*) indicates the normal cancerous cells and *green* (*lower*) treated cells, Plot of [SHP-2*] vs time. **b** *Blue color* (*upper*) indicates the normal cancerous cells and *green* (*lower*) treated cells, Plot of [PP1-STAT3C*] vs time

Figure 5a, b represents the concentration of complex (IL6-gp80- gp130-JAK)*2 –SHP2 in normal and treated condition. If the STAT3 component is inhibited, the level of (IL6-gp80- gp130-JAK)*2 –SHP2 complex will be reduced and will indeed affect the initiation of MAPK pathway and the JAK/STAT pathway. Reduction in the concentration of (IL6-gp80- gp130-JAK)*2 –SHP2 complex due to STAT3 inhibition, also affects the phosphorylated complex, (IL6-gp80- gp130-JAK)*2 –SHP2* and the SHP-2* component. These phosphorylated events form an important part of the MAPK pathway. Figure 6a represents the normal and treated condition of, (IL6-gp80- gp130-JAK)*2 –SHP2* and SHP-2* which shows the decrease in the level of concentration as compared to the normal condition and hampering the MAPK pathway.

### PP1 and PP2 complexes

**Fig. 7:**
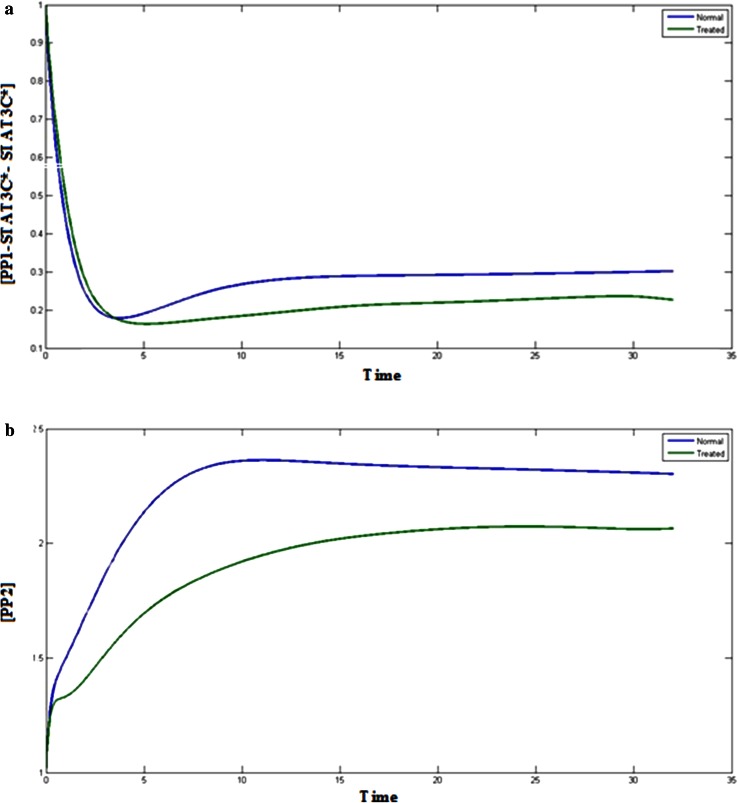
**a** *Blue color* (*upper*) indicates the normal cancerous cells and *green* (*lower*) treated cells, Plot of [PP1-STAT3C*- STAT3C*] vs time. **b** *Blue* *color* (*upper*) indicates the normal cancerous cells and *green* (*lower*) indicates the treated cells, Plot of [PP2] vs time

JAK-STAT signal transduction pathway is regulated by the two STAT3 phosphatases, PP1 and PP2. Theses phosphates influence the activation of STAT component in both the cytosol and the nucleus. Figures 6b and 7a represents the normal and treated condition of the PP1 complex, PP1-STAT3C* and PP1-STAT3C*- STAT3C*. Inhibition of STAT3 also influences. Inhibition of STAT3 component affects the PP1 and due to which the concentration of PP1 complexes are also reduced. If PP1 is affected, it will hamper the activation of STAT component then further the JAK/STAT pathway. Along with STAT3, PP2 also plays a crucial role in JAK/STAT signaling pathway. Figure 7b represents the behavior of PP2 with respect to time when the STAT3 is in normal and treated condition. It can be analyzed that level of PP2 in treated condition is reaching to the lower level as compared the normal condition at the end of the simulation. This shows that STAT3 is affecting the levels of PP2 in the pathway. PP2 deactivates the phosphorylated STAT3 dimer STAT3 N- STAT3 N* in the nucleus. This deactivation of STAT3 is necessary for the phosphorylation and dephosphorylation cycle of STAT3 in the JAK/STAT signaling pathway. If the STAT3 is inhibited, the level of PP2 will be decreased and it will not be able to deactivate the phosphorylated STAT3 dimer in the nucleus. This condition will affect the concentration of STAT3 in cytoplasm and STAT3 N*- STAT3 N* in the nucleus and will further reduce the efficiency of signaling process through JAK-STAT pathway and other cellular processes.

## Conclusion

In this work, mathematical models of all biochemical reactions of CML associated signaling pathways like JAK/STAT and MAPK have been generated. An attempt has been made to investigate the effect of inhibitors on particular receptor like STAT3 receptor in CML associated signaling pathways. Each step of pathways was converted into respective biochemical kinetics following mass action principle of michaelis–menten kinetic reactions. We proposed an effective optimization approach for estimating model parameters of the JAK/STAT and MAPK signaling pathways. *Fminsearch* function was used to minimize the error during simulation process. It generates observation data at different time interval together with the first order derivatives of the model parameters. The calculated parameters values were used to infer unknown parameters. Simulation results suggested that the proposed optimization method is effective, robust and reliable parameter estimation technique. Further the role of STAT3 has also been predicted in CML associated signaling pathways. Since the phosphorylation of STAT3 is crucial for signaling process of JAK/STAT and MAPK pathway, hence its inhibition affects other components of the pathways such as PP1, PP2, and SHP2. Inhibitory effect of the BP-5-087 ligand on STAT3 receptor was compared with the normal (untreated condition). It can be inferred from the simulation results that BP-5-087 can successfully reduce the progression of CML which is further in concurrence with the earlier reported in vitro study by Eiring et al. (2015). The proposed model may be utilized to analyze an overall impact of various inhibitors targeting the CML associated receptor proteins.

## Electronic supplementary material

Below is the link to the electronic supplementary material.

Supplementary material 1 (DOC 114 kb)
