# Supplementary material for: Effect of STAT3 inhibitor in chronic myeloid leukemia associated signaling pathway: a mathematical modeling, simulation and systems biology study
Source: 3 Biotech. 2016 Jan 27;6(1):40. doi: 10.1007/s13205-015-0357-7 (PMC4729759; doi:10.1007/s13205-015-0357-7)
Supplement: Supplementary file 1 — Supplementary material 1 (DOC 114 kb) [file 13205_2015_357_MOESM1_ESM.docx]

**Supplementary Material 1**

Modeled chemical kinetics of the whole pathway:

1. $gp80 + IL6 IL6-gp80$
2. $gp130 + JAKgp130-JAK$
3. $IL6-gp80 + gp130-JAKIL6-gp80-gp130-JAK$
4. $IL6-gp80-gp130-JAK + IL6-gp80-gp130-JAK (IL6-gp80-gp130-JAK)2$
5. $(IL6-gp80-gp130-JAK)2 (IL6-gp80-gp130-JAK)*2$
6. $(IL6-gp80-gp130-JAK)*2 + STAT3C (IL6-gp80-gp130-JAK)*2-STAT3C$
7. $(IL6-gp80-gp130-JAK)*2 -STAT3C (IL6-gp80-gp130-JAK)*2 + STAT3C*$
8. $(IL6-gp80-gp130-JAK)*2 + STAT3C* (IL6-gp80-gp130-JAK)*2-STAT3C*$
9. $STAT3C* + STAT3C*STAT3C*-STAT3C*$
10. $(IL6-gp80-gp130-JAK)*2 + SHP2 (IL6-gp80-gp130-JAK)*2 -SHP2$
11. $(IL6-gp80-gp130-JAK)*2 -SHP2 (IL6-gp80-gp130-JAK)2 + SHP2$
12. $PP1 + STAT3C*PP1-STAT3C*$
13. $PP1-STAT3C*PP1 + STAT3C$
14. $PP1 + STAT3C*-STAT3C*PP1-STAT3C*-STAT3C*$
15. $PP1-STAT3C*-STAT3C*PP1 + STAT3C-STAT3C*$
16. $STAT3C + STAT3C*STAT3C-STAT3C*$
17. $STAT3C*-STAT3C*STAT3N*-STAT3N*$
18. $STAT3N*-STAT3N*STAT3N* + STAT3N*$
19. $PP2 + STAT3N*PP2-STAT3N*$
20. $PP2-STAT3N*PP2 + STAT3N$
21. $PP2 + STAT3N*-STAT3N*PP2-STAT3N*-STAT3N*$
22. $PP2-STAT3N*-STAT3N*PP2 + STAT3N-STAT3N*$
23. $STAT3N-STAT3N*STAT3N + STAT3N*$
24. $STAT3NSTAT3C$
25. $STAT3N*-STAT3N*mRNA-SOCS3N$
26. $mRNA-SOCS3NmRNA-SOCS3C$
27. $mRNA-SOCS3CSOCS3$
28. $SOCS3 + (IL6-gp80-gp130-JAK)*2 (IL6-gp80-gp130-JAK)*2 -SOCS3$
29. $mRNA-SOCS3CmRNA-SOCS3C$
30. $SOCS3 SOCS3$
31. $(IL6-gp80-gp130-JAK)*2-SOCS3 SOCS3 + (IL6-gp80-gp130-JAK)2$
32. $(IL6-gp80-gp130-JAK)*2-SHP2 (IL6-gp80-gp130-JAK)*2-SHP2*$
33. $(IL6-gp80-gp130-JAK)*2-SHP2* + Grb2 (IL6-gp80-gp130-JAK)*2-SHP2*-Grb2$
34. $(IL6-gp80-gp130-JAK)*2-SHP2*-Grb2 + SOS(IL6-gp80-gp130-JAK)*2-SHP2*-Grb2-SOS$
35. $(IL6-gp80-gp130-JAK)*2-SHP2*-Grb2-SOS + Ras-GDP(IL6-gp80-gp130-JAK)*2-SHP2*-Grb2-SOS-Ra-GDP$
36. $(IL6-gp80-gp130-JAK)*2-SHP2*-Grb2-SOS-Ra-GDP (IL6-gp80-gp130-JAK)*2-SHP2*-Grb2-SOS + Ras-GTP$
37. $Raf + Ras-GTPRaf-Ras-GTP$
38. $Raf + Ras-GTPRaf* + Ras-GTP*$
39. $Ras-GTP* + (IL6-gp80-gp130-JAK)*2-SHP2*-Grb2-SOS (IL6-gp80-gp130-JAK)*2-SHP2*-Grb2-SOS-Ras-GTP$
40. $(IL6-gp80-gp130-JAK)*2-SHP2*-Grb2-SOS-Ras-GTP (IL6-gp80-gp130-JAK)*2-SHP2*-Grb2-SOS + Ras-GDP$
41. $(IL6-gp80-gp130-JAK)*2-SHP2*-Grb2-SOS (IL6-gp80-gp130-JAK)*2 + SHP2*-Grb2-SOS$
42. $SHP2*-Grb2-SOSGrb2-SOS + SHP2*$
43. $Grb2-SOSGrb2 + SOS$
44. $SHP2*SHP2$
45. $(IL6-gp80-gp130-JAK)*2-SHP2*(IL6-gp80-gp130-JAK)*2 + SHP2*$
46. $SHP2* + Grb2 SHP2*-Grb2$
47. $(IL6-gp80-gp130-JAK)*2-SHP2*-Grb2 SHP2*-Grb2 + (IL6-gp80-gp130-JAK)*2$
48. $SHP2*-Grb2 + SOSSHP2*-Grb2-SOS$
49. $(IL6-gp80-gp130-JAK)*2-SHP2* + Grb2-SOS(IL6-gp80-gp130-JAK)*2-SHP2*-Grb2-SOS$
50. $Raf* + Phosp1 Raf*-Phosp1$
51. $Raf*-Phosp1 Raf + Phosp1$
52. $MEK + Raf*MEK-Raf*$
53. $MEK-Raf*MEK-P + Raf*$
54. $MEK-P + Raf*MEK-P-Raf*$
55. $MEK-P-Raf*MEK-PP + Raf*$
56. $MEK-PP + Phosp2 MEK-PP-Phosp2$
57. $MEK-PP-Phosp2 MEK-P + Phosp2$
58. $MEK-P + Phosp2 MEK-P-Phosp2$
59. $MEK-P-Phosp2 MEK + Phosp2$
60. $ERK + MEK-PPERK-MEK-PP$
61. $ERK-MEK-PPERK-P + MEK-PP$
62. $ERK-P + MEK-PPERK-P-MEK-PP$
63. $ERK-P-MEK-PPERK-PP + MEK-PP$
64. $ERK-PP + Phosp3 ERK-PP-Phosp3$
65. $ERK-PP-Phosp3 ERK-PP + Phosp3$
66. $ERK-PP + Phosp3 ERK-P-Phosp3$
67. $ERK-P-Phosp3 ERK + Phosp3$

**Supplementary Material 2**

**Table :** State variables of the model and initial concentration of all substrates.

| **Name** | **Component** | **Experimental value (nM)** |
| --- | --- | --- |
| U | IL6 | 3.83 (100 ng/ml) |
| x1 | gp80 | 8 |
| x2 | IL6-gp80 | 0 |
| x3 | gp130 | 0.8 |
| x4 | JAK | 12 |
| x5 | gp130-JAK | 0 |
| x6 | IL6-gp80- gp130-JAK | 0 |
| x7 | (IL6-gp80- gp130-JAK)2 | 0 |
| x8 | (IL6-gp80- gp130-JAK)*2 | 0 |
| x9 | STAT3C | 1000 |
| x10 | (IL6-gp80- gp130-JAK)*2 -STAT3C | 0 |
| x11 | STAT3C* | 0 |
| x12 | (IL6-gp80- gp130-JAK)*2 -STAT3C* | 0 |
| x13 | STAT3C* -STAT3C* | 0 |
| x14 | SHP2 | 100 |
| x15 | (IL6-gp80- gp130-JAK)*2 –SHP2 | 0 |
| x16 | PP1 | 50 |
| x17 | PP1-STAT3C* | 0 |
| x18 | PP1-STAT3C*- STAT3C* | 0 |
| x19 | STAT3C -STAT3C* | 0 |
| x20 | STAT3N*- STAT3N* | 0 |
| x21 | STAT3N* | 0 |
| x22 | PP2 | 60 |
| x23 | PP2-STAT3N* | 0 |
| x24 | STAT3N | 0 |
| x25 | PP2-STAT3N*- STAT3N* | 0 |
| x26 | STAT3N- STAT3N* | 0 |
| x27 | mRNA-SOCS3N | 0 |
| x28 | mRNA-SOCS3C | 0 |
| x29 | SOCS3 | 0 |
| x30 | (IL6-gp80- gp130-JAK)*2 –SOCS3 | 0 |
| x31 | (IL6-gp80- gp130-JAK)*2 –SHP2* | 0 |
| x32 | Grb2 | 85 |
| x33 | (IL6-gp80- gp130-JAK)*2 –SHP2*-Grb2 | 0 |
| x34 | SOS | 34 |
| x35 | (IL6-gp80- gp130-JAK)*2 –SHP2*-Grb2-SOS | 0 |
| x36 | Ras-GDP | 19000 |
| x37 | (IL6-gp80- gp130-JAK)*2 –SHP2*-Grb2-SOS-Ras-GDP | 0 |
| x38 | Ras-GTP | 0 |
| x39 | Raf | 67 |
| x40 | Raf-Ras-GTP | 0 |
| x41 | Raf* | 0 |
| x42 | Ras-GTP* | 0 |
| x43 | (IL6-gp80- gp130-JAK)*2 –SHP2*-Grb2-SOS-Ras-GTP | 0 |
| x44 | SHP2*-Grb2-SOS | 0 |
| x45 | Grb2- SOS | 0 |
| x46 | SHP2* | 0 |
| x47 | SHP2*-Grb2 | 0 |
| x48 | Phosp1 | 67 |
| x49 | Raf*- Phosp1 | 0 |
| x50 | MEK | 41667 |
| x51 | MEK-Raf* | 0 |
| x52 | MEK-P | 0 |
| x53 | MEK-P-Raf* | 0 |
| x54 | MEK-PP | 0 |
| x55 | Phosp2 | 67 |
| x56 | MEK-PP-Phosp2 | 0 |
| x57 | MEK-P-Phosp2 | 0 |
| x58 | ERK | 35000 |
| x59 | ERK- MEK-PP | 0 |
| x60 | ERK- P | 0 |
| x61 | ERK-P- MEK-PP | 0 |
| x62 | ERK- PP | 0 |
| x63 | Phosp3 | 16667 |
| x64 | ERK-PP- Phosp3 | 0 |
| x65 | ERK-P- Phosp3 | 0 |

**Supplementary Material 3: ODE equations of the entire pathways (JAK/STAT and MAPK)**

$\frac{dx1}{dt}= 0$

$\frac{dx2}{dt}= kf1*x1*u-kr1*x2 -kf3*x2*x5 + kr3*x6$

$\frac{dx3}{dt}= 0$

$\frac{dx4}{dt}= -kf2*x3*x4 + kr2*x5$

$\frac{dx5}{dt}= kf2*x3*x4 -kr2*x5 -kf3*x2*x5 + kr3*x6$

$\frac{dx6}{dt}= kf3*x2*x5 -kr3*x6 - 2*kf4*x6*x6 + 2*kr4*x7$

$\frac{dx7}{dt}= kf4*x6*x6 -kr4*x7 -k5*x7 + k11*x15 + k31*x30$

$\frac{dx8}{dt}= k5*x7 -kf6*x8*x9 + kr6*x10 + k7*x10 -kf8*x8*x11 + kr8*x12 -kf10*x8*x14 + kr10*x15 -kf28*x29*x8 + kr28*x30 + kf41*x35 -kr41*x8*x44 + kf45*x31 -kr45*x8*x46 + kf47*x33 -kr47*x47*x8$

$\frac{dx9}{dt}= -kf6*x8*x9 + kr6*x10 + k13*x17 -kf16*x9*x11 + kr16*x19 + k24*x24$

$\frac{dx10}{dt}= kf6*x8*x9 -kr6*x10 -k7*x10$

$\frac{dx11}{dt}= k7*x10 -kf8*x8*x11 + kr8*x12 - 2*kf9*x11*x11 + 2*kr9*x13 -kf12*x16*x11 + kr12*x17 -kf16*x9*x11 + kr16*x19$

$\frac{dx12}{dt}= kf8*x8*x11 -kr8*x12$

$\frac{dx13}{dt}= kf9*x11*x11 -kr9*x13 -kf14*x16*x13 + kr14*x18 -k17*x13$

$\frac{dx14}{dt}= -kf10*x8*x14 + kr10*x15 + k11*x15 + vm*\frac{x46}{km + x46}$

$\frac{dx15}{dt}= kf10*x8*x14 -kr10*x15 -k11*x15 -kf32*x15 + kr32*x31$

$\frac{dx16}{dt}= -kf12*x16*x11 + kr12*x17 + k13*x17 -kf14*x16*x13 + kr14*x18 + k15*x18$

$\frac{dx17}{dt}= kf12*x16*x11 -kr12*x17 -k13*x17$

$\frac{dx18}{dt}= kf14*x16*x13 -kr14*x18 -k15*x18$

$\frac{dx19}{dt}= k15*x18 + kf16*x9*x11 -kr16*x19$

$\frac{dx20}{dt}= k17*x13 -kf18*x20 + kr18*x21*x21 -kf21*x22*x20 + kr21*x25$

$\frac{dx21}{dt}= 2*kf18*x20 - 2*kr18*x21*x21 -kf19*x22*x21 + kr19*x23 + kr23*x26 -kf23*x24*x21$

$\frac{dx22}{dt}= -kf19*x22*x21 + kr19*x23 + k20*x23 -kf21*x22*x20 + kr21*x25 + k22*x25$

$\frac{dx23}{dt}= kf19*x22*x21 -kr19*x23 -k20*x23$

$\frac{dx24}{dt}= k20*x23 + kr23*x26 -kf23*x24*x21 -k24*x24$

$\frac{dx25}{dt}= kf21*x22*x20 -kr21*x25 -k22*x25$

$\frac{dx26}{dt}= k22*x25 -kr23*x26 + kf23*x24*x21$

$\frac{dx27}{dt}= k25a*\frac{x20}{k25b + x20}-k26*x27$

$\frac{dx28}{dt}= k26*x27 -k29*x28$

$$\frac{dx29}{dt}= k27*x28 -kf28*x29*x8 + kr28*x30 -k30*x29 + k31*x30$$

$\frac{dx30}{dt}= kf28*x29*x8 -kr28*x30 -k31*x30$

$\frac{dx31}{dt}= kf32*x15 -kr32*x31 -kf33*x31*x32 + kr33*x33 -kf45*x31 + kr45*x8*x46 -kf49*x31*x45 + kr49*x35$

$\frac{dx32}{dt}= -kf33*x31*x32 + kr33*x33 + kf43*x45 -kr43*x32*x34 -kf46*x46*x32 + kr46*x47$

$\frac{dx33}{dt}= kf33*x31*x32 -kr33*x33 -kf34*x33*x34 + kr34*x35 -kf47*x33 + kr47*x47*x8$

$\frac{dx34}{dt}= -kf34*x33*x34 + kr34*x35 + kf43*x45 -kr43*x32*x34 -kf48*x47*x34 + kr48*x44$

$\frac{dx35}{dt}= kf34*x33*8x34 -kr34*x35 -kf35*x35*x36 + kr35*x37 + kf36*x37 -kr36*x37*x38 -kf39*x42*x35 + kr39*x43 + kf40*x43 -kr40*x35*x36 -kf41*x35 + kr41*x8*x44 + kf49*x31*x45 -kr49*x35$

$\frac{dx36}{dt}= -kf35*x35*x36 + kr35*x37 + kf40*x43 -kr40*x35*x36$

$\frac{dx37}{dt}= kf35*x35*x36 -kr35*x37 -kf36*x37 + kr36*x35*x38$

$\frac{dx38}{dt}= kf36*x37 -kr36*x35*x38 -kf37*x39*x38 + kr36*x40$

$\frac{dx39}{dt}= -kf37*x39*x38 + kr37*x40 + k51*x49$

$\frac{dx40}{dt}= kf37*x39*x38 -kr37*x40 -kf38*x40 + kr38*x41*x42$

$\frac{dx41}{dt}= kf38*x40 -kr38*x41*x42 -kf50*x41*x48 + kr50*x49 -kf52*x50*x41 + kr52*x51 + k53*x51 -kf54*x52*x41 + kr54*x53 + k55*x53$

$\frac{dx42}{dt}= kf38*x40 -kr38*x41*x42 -kf39*x42*x35 + kr39*x43$

$\frac{dx43}{dt}= kf39*x42*x35 -kr39*x43 -kf40*x43 + kr40*x35*x36$

$\frac{dx44}{dt}= kf41*x35 -kr41*x8*x44 -kf42*x44 + kr42*x45*x46 + kf48*x47*x34 -kr48*x44$

$\frac{dx45}{dt}= kf42*x44 -kr42*x45*x46 -kf43*x45 + kr43*x32*x34 -kf49*x31*x45 + kr49*x35$

$\frac{dx46}{dt}= kf42*x44 -kr42*x45*x46 -vm*\frac{x46}{km + x46}+ kf45*x31 -kr45*x8*x46 -kf46*x46*x32 + kr46*x47$

$\frac{dx47}{dt}= kf46*x46*x32 -kr46*x47 + kf47*x33 -kr47*x47*x8 -kf48*x47*x34 + kr48*x44$

$\frac{dx48}{dt}= -kf50*x41*x48 + kr50*x49 + k51*x49$

$\frac{dx49}{dt}= kf50*x41*x48 -kr50*x49 -k51*x49$

$\frac{dx50}{dt}= -kf52*x50*x41 + kr52*x51 + k59*x57$

$\frac{dx51}{dt}= kf52*x50*x41 -kr52*x51 -k53*x51$

$\frac{dx52}{dt}= k53*x51 -kf54*x52*x41 + kr54*x53 + k57*x56 -kf58*x52*x55 + kr58*x57$

$\frac{dx53}{dt}= kf54*x52*x41 -kr54*x53 -k55*x53$

$\frac{dx54}{dt}= k55*x53 -kf56*x54*x55 + kr56*x56 -kf60*x58*x54 + kr60*x59 + k61*x59 -kf62*x60*x54 + kr62*x61 + k63*x61$

$\frac{dx55}{dt}= -kf56*x54*x55 + kr56*x56 + k57*x56 -kf58*x52*x55 + kr58*x57 + k59*x57$

$\frac{dx56}{dt}= kf56*x54*x55 -kr56*x56 -k57*x56$

$\frac{dx57}{dt}= kf58*x52*x55 -kr58*x57 -k59*x57$

$\frac{dx58}{dt}= -kf60*x58*x54 + kr60*x59 + k67*x65$

$\frac{dx59}{dt}= kf60*x58*x54 -kr60*x59 -k61*x59$

$\frac{dx60}{dt}= k61*x59 -kf62*x60*x54 + kr62*x61 + k65*x64 -kf66*x60*x63 + kr66*x65$

$\frac{dx61}{dt}= kf62*x60*x54 -kr62*x61 -k63*x61$

$\frac{dx62}{dt}= k63*x61 -kf64*x62*x63 + kr64*x64$

$\frac{dx63}{dt}= -kf64*x62*x63 + kr64*x64 + k65*x64 - kf66*x60*x63 + kr66*x65 + k67*x65$

$\frac{dx64}{dt}= kf64*x62*x63 - kr64*x64 - k65*x64$

$\frac{dx65}{dt}= kf66*x60*x63 - kr66*x65 - k67*x65$

Where, kfi and kri are the rate constants of forward and backward chemical reactions of mass action kinetics and kmi, kvi are the rate constant of michaelis-menten kinetics .
